# Is the central‐marginal hypothesis a general rule? Evidence from three distributions of an expanding mangrove species, *Avicennia germinans* (L.) L

**DOI:** 10.1111/mec.15365

**Published:** 2020-02-14

**Authors:** John Paul Kennedy, Richard F. Preziosi, Jennifer K. Rowntree, Ilka C. Feller

**Affiliations:** ^1^ Smithsonian Marine Station Smithsonian Institution Fort Pierce FL USA; ^2^ Department of Natural Sciences Faculty of Science and Engineering, Ecology and Environment Research Centre Manchester Metropolitan University Manchester UK; ^3^ Smithsonian Environmental Research Center Smithsonian Institution Edgewater MD USA

**Keywords:** abundant‐centre distribution, central‐periphery hypothesis, coastal species, functional traits, genetic diversity, range limits

## Abstract

The central‐marginal hypothesis (CMH) posits that range margins exhibit less genetic diversity and greater inter‐population genetic differentiation compared to range cores. CMH predictions are based on long‐held “abundant‐centre” assumptions of a decline in ecological conditions and abundances towards range margins. Although much empirical research has confirmed CMH, exceptions remain almost as common. We contend that mangroves provide a model system to test CMH that alleviates common confounding factors and may help clarify this lack of consensus. Here, we document changes in black mangrove (*Avicennia germinans*) population genetics with 12 nuclear microsatellite loci along three replicate coastlines in the United States (only two of three conform to underlying “abundant‐centre” assumptions). We then test an implicit prediction of CMH (reduced genetic diversity may constrain adaptation at range margins) by measuring functional traits of leaves associated with cold tolerance, the climatic factor that controls these mangrove distributional limits. CMH predictions were confirmed only along the coastlines that conform to “abundant‐centre” assumptions and, in contrast to theory, range margin *A. germinans* exhibited functional traits consistent with greater cold tolerance compared to range cores. These findings support previous accounts that CMH may not be a general rule across species and that reduced neutral genetic diversity at range margins may not be a constraint to shifts in functional trait variation along climatic gradients.

## 1 INTRODUCTION

Understanding factors that control species distributional limits is a central objective in ecology (Gaston, 2009; Hardie & Hutchings, 2010), and necessary insight to better predict responses to climate change (Chen, Hill, Ohlemüller, Roy, & Thomas, 2011). The basis of many theories on distributional limits are long‐held “abundant‐centre” assumptions, that species experience optimal conditions and highest abundances in the central distributional core and lowest abundances towards range limits, where marginal conditions impede population growth and survival (Sagarin & Gaines, 2002). While range cores are generally stable, range margins can be quite mobile as species expand or contract in response to environmental changes (Sexton, McIntyre, Angert, & Rice, 2009).

In line with these innate differences, the central‐marginal hypothesis (CMH), also called the central‐periphery hypothesis (Pironon et al., 2017), posits that range margins exhibit lower intrapopulation genetic diversity and higher interpopulation genetic differentiation compared to range cores because of reduced population sizes and greater isolation (Eckert, Samis, & Lougheed, 2008). Much empirical research has confirmed CMH, but exceptions remain almost as common (Eckert et al., 2008; Pironon et al., 2017). Lack of consensus could be the result of numerous factors, including interspecific life history differences (Araújo, Serrão, Sousa‐Pinto, & Åberg, 2011), confounding effects of latitude (Guo, 2012), variation in past distributional fluctuations (Nadeau et al., 2015), or simply the intrinsic difficulty of defining range core and margin for many species (Sagarin, Gaines, & Gaylord, 2006). To test CMH, a common approach is to identify the range core as the geographical centre of a species range, based on the theory's underlying “abundant‐centre” assumptions (i.e., decline in ecological conditions and abundances towards range margins). However, this assumed pattern occurs much less often than previously expected (Sagarin & Gaines, 2002; Santini, Pironon, Maiorano, & Thuiller, 2019). Quantitative approaches have addressed this issue with evidence‐based estimates of range centre considering species biology (Schwartz, Mills, Ortega, Ruggiero, & Allendorf, 2003), climatic suitability (Lira‐Noriega & Manthey, 2014; Micheletti & Storfer, 2015), and genetic differences (Griffin & Willi, 2014); and have demonstrated that disentangling the relative effects of geographic, ecological, and historical gradients is often difficult when interpreting patterns across broad spatial scales (Pironon, Villellas, Morris, Doak, & García, 2015). Another means to achieve greater insight into the generality and implications of CMH would be to identify model systems that naturally conform to “abundant‐centre” assumptions and provide a test of this theory with less influence of confounding factors.

Coastal species provide ideal systems to test many large‐scale ecological theories because of their essentially one‐dimensional, and often widespread, distributions (Sagarin et al., 2006). We contend that mangroves, an assortment of (sub)tropical intertidal tree and shrub species, provide an ideal model system to test CMH. Mangrove distributions are easily defined because of their restriction to narrow intertidal zones (Tomlinson, 1986), and are anchored in the tropics where these plants reach their highest abundances and experience favourable climatic conditions (Spalding, Kainuma, & Collins, 2010). Mangrove abundance and species richness decrease towards poleward range limits, as climatic variables (i.e., temperature, precipitation) become more marginal (Osland, Feher, et al., 2017). Range cores generally remain stable, unless impacted by stochastic weather events (e.g., Smith, Robblee, Wanless, & Doyle, 1994) or anthropogenic changes (Valiela, Bowen, & York, 2001); whereas range limits are highly mobile due to climatic thresholds specific to individual geographic regions (Cavanaugh et al., 2018; Osland, Feher, et al., 2017).

**Figure 1:**
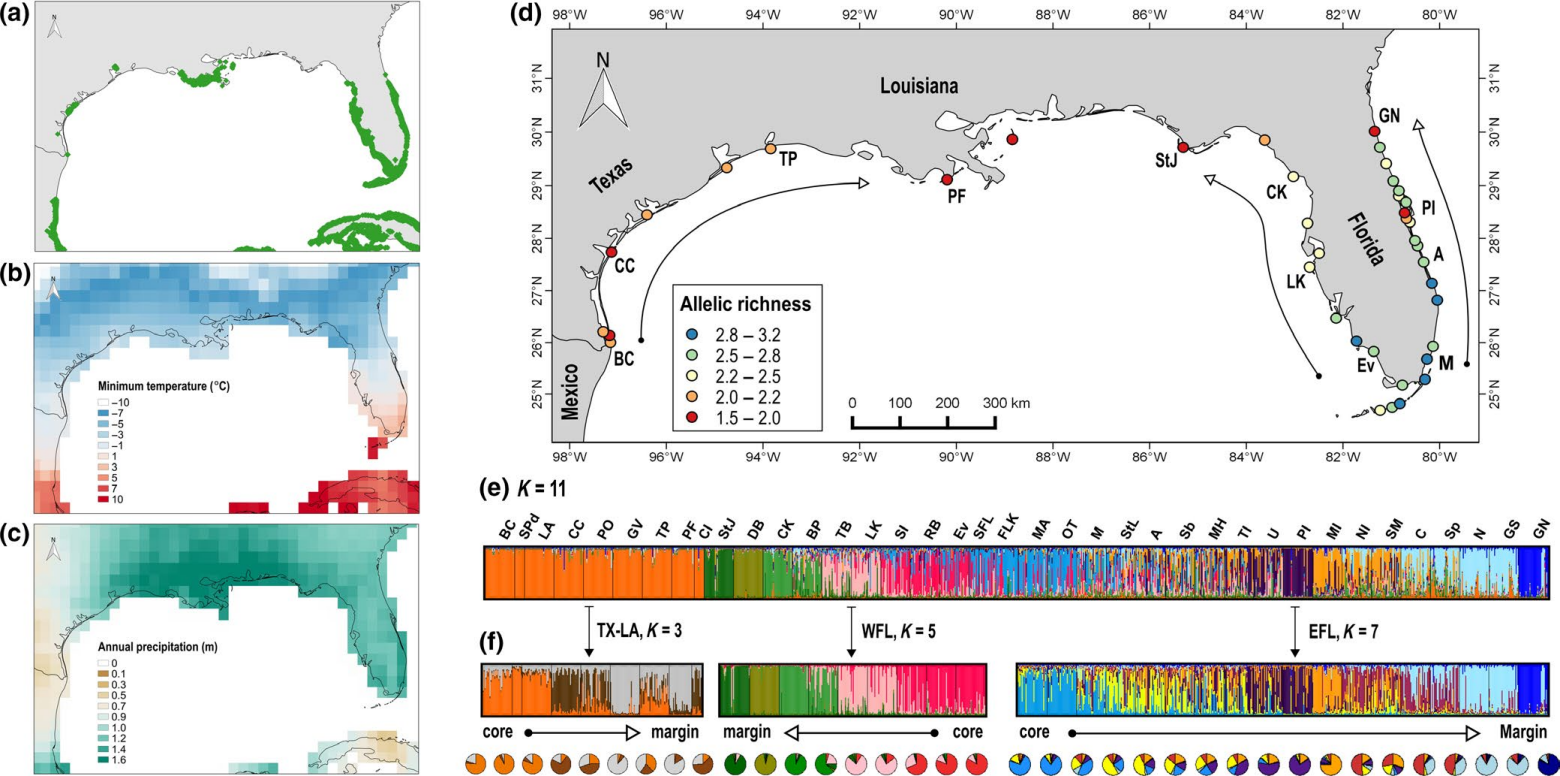
Evaluation of the central‐marginal hypothesis (CMH) in *Avicennia germinans* from three distribution ranges in the United States (USA): Texas‐Louisiana (TX‐LA) does not conform to the underlying “abundant‐centre” assumptions of this theory; West Florida (WFL) and East Florida (EFL) conform to these underlying assumptions. (a) Mangrove distribution in the USA and neighbouring countries (Giri et al., 2011). (b, c) Regional climatic factors that control mangrove abundances and distributional limits: (b) mean annual minimum temperature (°C) and (c) mean annual precipitation (m), both from 1980–2017. (d) Collection sites and neutral genetic diversity along TX‐LA, WFL, and EFL (from left to right). Arrows show core to margin along each distribution range. (e) Genetic structure along the entire USA distributional range estimated in STRUCTURE 2.3 based on changes in ln Pr(*X*|*K*) (*K* = 11 population clusters). Collection sites are shown from west to east and strategic site codes are included in panel d to aid visualization (Note: in panel d, collection site PI corresponds to the adjacent red dot). (f) Subsequent genetic structure along each of the three distribution ranges separately from core to margin (TX‐LA, *K* = 3; WFL, *K* = 5; EFL, *K* = 7), with averaged assignments for each collection site presented as pie charts. Refer to Table S1 for site codes [Colour figure can be viewed at http://www.wileyonlinelibrary.com/]

Black mangrove, *Avicennia germinans* (L.) L., is widespread throughout the Neotropics and the predominant mangrove species at range margins in the United States (USA) (Lonard, Judd, Summy, DeYoe, & Stalter, 2017), in part because of its greater freeze tolerance compared to co‐occurring mangrove species (Cavanaugh et al., 2015). In the USA, *A. germinans* is present along the coastlines of Texas‐Louisiana, West Florida, and East Florida; three natural replicates of core to margin distribution ranges (Figure 1). This system naturally controls for many common confounding factors, with a single, widespread model species and three coastlines along a similar latitudinal gradient (~26–30°N). We can also presume that these three coastlines experienced similar historical distributional fluctuations, as the present‐day USA mangrove distribution is thought to be the product of complete eradication at the Last Glacial Maximum, with retraction towards the equator, and subsequent Post‐Pleistocene recolonization (Sherrod & McMillan, 1985; also see descriptions in Osland et al., 2018; Rogers & Krauss, 2018; Saintilan, Wilson, Rogers, Rajkaran, & Krauss, 2014).

Although these three coastlines share many commonalities, there is a strong dichotomy between climatic factors controlling mangrove distributions in Texas compared to neighbouring Louisiana, and to Florida. Mangrove abundance and distributional limits in Florida and Louisiana are controlled by latitudinal gradients in minimum winter temperatures, whereas inverse latitudinal gradients in both minimum temperatures and precipitation control mangroves across Texas (Cavanaugh et al., 2018; Osland, Feher, et al., 2017) (Appendix S1 details this climatic information). Mangroves in the USA are most abundant in South Florida (with an assemblage of three principal species), transition into a mangrove‐salt marsh ecotone along both Florida coastlines, and are eventually replaced by salt marsh as freeze events become more common (Kangas & Lugo, 1990) (Figure 1a,b). In contrast, Texas mangroves (essentially only *A. germinans*) are far less abundant, without a continuous distribution, and mostly restricted to three distributional centres with the nearest continuous mangrove forest in Mexico, approximately 300 km south of the southernmost Texas mangroves (Guo, Zhang, Lan, & Pennings, 2013; Sherrod & McMillan, 1981) (Figure 1a). Hypersaline conditions that exceed physiological thresholds are common along South and Central Texas, and limit mangrove presence and abundance (Gabler et al., 2017; Osland et al., 2016); whereas rainfall increases along North Texas, and into adjacent Louisiana where *A. germinans* reach comparatively higher abundances (Osland et al., 2016) (Figure 1a,c).

Southern range core mangroves are relatively stable in Florida (but see Ross, Ruiz, Sah, & Hanan, 2009; Zhang, Thapa, Ross, & Gann, 2016) and Texas (but see Lonard & Judd, 1991) compared to highly‐mobile range margins (Cavanaugh et al., 2018). Periodic extreme freeze events lead to declines in mangrove cover at USA range margins and a cyclical pattern of expansion and contraction over time across the region (Giri & Long, 2016; Osland, Day, et al., 2017; Rodriguez, Feller, & Cavanaugh, 2016; Rogers & Krauss, 2018). An absence of extreme freezes since the late 1980s is linked to ongoing, rapid range expansion of *A. germinans* into salt marsh habitat at all USA northern limits, in Texas (Armitage, Highfield, Brody, & Louchouarn, 2015; Everitt, Yang, Judd, & Summy, 2010), Louisiana (Osland, Day, et al., 2017), West Florida (Saintilan et al., 2014; Stevens, Fox, & Montague, 2006), and East Florida (Cavanaugh et al., 2014; Rodriguez et al., 2016), with further proliferation at, and expansion past, these range margins forecast with climate change (Cavanaugh et al., 2015; Osland, Day, et al., 2017; Osland, Enwright, Day, & Doyle, 2013).

This wealth of previous research demonstrates that USA mangroves simplify tests of CMH as parallel gradients in latitude, ecological marginality (in terms of minimum temperatures), and postglacial recolonization exist along each of these three distribution ranges. West and East Florida *A. germinans* also conform to the underlying “abundant‐centre” assumptions of CMH, whereas Texas‐Louisiana conspecifics do not (Table 1). Instead, the entire Texas‐Louisiana distribution resembles a range margin, with the true range core (i.e., optimal ecological conditions and highest abundances) located farther south in Mexico. Texas‐Louisiana, therefore, provides a test of CMH when “abundant‐centre” assumptions are not met, a proposed reason for limited consensus regarding CMH (Eckert et al., 2008; Pironon et al., 2017).

**Table 1:** Results summary of central‐marginal hypothesis (CMH) underlying “abundant‐centre” assumptions, explicit predictions, and an implicit prediction along three *Avicennia germinans* distribution ranges in the United States: Texas‐Louisiana (TX‐LA), West Florida (WFL) and East Florida (EFL)

	TX‐LA	WFL	EFL
CMH “abundant‐centre” assumptions			
Decline in ecological conditions towards range margin	No	Yes	Yes
Decline in abundances towards range margin	No	Yes	Yes
CMH explicit predictions			
Reduced intrapopulation genetic diversity towards range margin	No	Yes	Yes
Increased interpopulation genetic differentiation towards range margin	No	Yes	Yes
CMH implicit prediction			
Constrained adaptation to environmental conditions towards range margin^a^	No	No	No

a To test this implicit prediction, we evaluated changes in functional traits of leaves associated with cold tolerance.

An implicit prediction of CMH is that limited adaptive genetic diversity at range margins can reduce evolutionary potential and constrain adaptation to environmental conditions at the distributional limit, which can impede further expansion (Bridle & Vines, 2007). Theoretical research predicts that range dynamics are controlled by the interactive effects of gene flow and genetic drift, and also the effect of genetic variation on trait expression (Connallon & Sgrò, 2018 and citations within). This last factor is of particular importance because genetic diversity is most often measured with neutral molecular markers, that may not reflect variation in adaptive genetic diversity of ecological significance (Gaston, 2009). Empirical research, although limited, suggests that reduced neutral genetic variation at range margins does not translate into reduced ecologically‐relevant trait variation compared to range cores (Abeli, Gentili, Mondoni, Orsenigo, & Rossi, 2014; Kawecki, 2008; Pironon et al., 2017). Hence, an integration of measures of neutral genetic variation and of context‐specific trait variation will improve our understanding of the potential implications of CMH.

Climatic factors shaping mangrove distributions also influence mangrove morphological traits and physiological adaptations (Clough, 1992). USA mangrove canopy heights decrease along temperature and precipitation gradients towards range margins (Feher et al., 2017). At these margins, *A. germinans* exhibit variation in xylem vessel architecture (Madrid, Armitage, & López‐Portillo, 2014; Stuart, Choat, Martin, Holbrook, & Ball, 2007) and leaf traits (Cook‐Patton, Lehmann, & Parker, 2015) consistent with greater freeze tolerance compared to range‐core conspecifics. Here, we measured functional traits of leaves associated with cold tolerance. Functional traits are attributes that can influence establishment, survival and fitness (Pérez‐Harguindeguy et al., 2013). Freeze‐resistant plant species exhibit conservative leaf traits better suited to tolerate stress, including reduced leaf length and width (Jordan & Smith, 1995), reduced leaf area (Pérez‐Harguindeguy et al., 2013), and reduced specific leaf area (Poorter, Niinemets, Poorter, Wright, & Villar, 2009); although similar traits are also consistent with drought resistance (Knight & Ackerly, 2003).

In this study, we measured variation in both population genetics and cold‐stress associated functional traits of leaves across the entire USA distribution of *A. germinans*. We tested explicit genetic predictions of CMH and an implicit prediction along three replicate core to margin distribution ranges, two of which conform to the underlying “abundant‐centre” assumptions of this theory and a third that does not (Table 1 outlines CMH assumptions and predictions). Along each of the three distribution ranges, we asked: (a) Does neutral intrapopulation genetic diversity decrease towards range margins?; (b) Does interpopulation genetic differentiation increase towards range margins?; (c) Do functional traits of leaves exhibit changes consistent with greater cold tolerance towards range margins?

## 2 MATERIALS AND METHODS

### 2.1 Range classification

USA *A. germinans* only represent the northern extent of this species' entire distribution, with the true range centre closer to the equator. However, radiating out from this centre, the range core remains relatively continuous until range limits are defined by abrupt ecological thresholds (Osland et al., 2016). As such, USA *A. germinans* provide three extensions of this more‐equatorial range core that eventually transition into climate‐sensitive northern range margins (Figure S1). We defined range core as the most southern populations and all areas progressively northward where either pure mangrove exists or mangroves are the dominant foundation species. We used published descriptions to define range margin based on latitude, abundance, and population stability (Table S1 details collection site classifications). USA mangroves are replaced by salt marsh at approximately 29°N, where isolated, low‐abundance mangrove stands exist in a salt marsh‐dominated landscape (Spalding et al., 2010). Range‐margin sites in East Florida (29.4–30.0°N) and West Florida (29.1°N–29.8°N) are isolated from the continuous range core and are documented *A. germinans* range limits (Kangas & Lugo, 1990), with climate‐driven fluctuations in abundance over time in both areas (Montague & Odum, 1997; Rodriguez et al., 2016). Range‐margin sites in Texas and Louisiana (29.1°N–29.8°N) are also documented *A. germinans* range limits with evidence of fluctuations in abundance over time (Osland, Day, et al., 2017; Sherrod & McMillan, 1981), including complete mangrove die‐back at Texas Point (29.6°N; code: TP) (Sherrod & McMillan, 1981) where only five trees were identified in 2010 (Guo et al., 2013).

### 2.2 Sample collection: Genetic and functional trait analyses

Leaves were collected from a total of 1,083 *A. germinans* trees from 41 collection sites across this species' entire USA distributional range (Table S1; Figure 1). We collected samples along East Florida (EFL) in January 2015, along West Florida (WFL) in September–October 2015, and along Texas and Louisiana (TX‐LA) in October 2015. Samples for two sites (code: TB, SFL) were obtained from preserved leaves collected in 2011. For densely‐populated sites, sampled trees were located at least 20 m apart; whereas, for sparsely‐populated range‐margin sites, sampled trees were located as far apart as possible (generally at least 10 m) in an attempt to sample the entire site. We sampled a greater number of collection sites along EFL due to the complexity of the lagoon system along this coastline, which consists of three interconnected water bodies. This more comprehensive sampling strategy ensured that the entire system was characterized, including one site (code: PI) that has undergone substantial land modifications during conversion into a conservation area. We sampled all major distributional centres along WFL and TX‐LA. For genetic analyses, we collected leaves from 30 trees per site (except for site PF, *n* = 23). Fewer samples (*n* = 9–11) were collected opportunistically at additional sites between 2015 and 2016, and were used in region‐wide analyses (Figure 1d–e), but were not included in the subsequent genetic and functional trait CMH prediction tests. As such, to test CMH predictions, the EFL distribution range included 18 collection sites (25.6°N–30.0°N), WFL included nine sites (25.8°N–29.8°N), and TX‐LA included seven sites (26.0°N–29.6°N), with South Florida collections from the Everglades and Florida Keys not included in prediction tests (Table S1).

We measured functional traits for all sites with *n* ≥ 23 genetic samples, except for site TB where samples had been collected in 2011. We collected 10 leaves from each of ≥10 trees per site for functional trait measurements, a subset of the same trees sampled for genetic analyses. Within each site, we sampled mature (reproductive) trees that were all approximately the same height. Each of the 10 leaves per tree was from the most fully‐expanded, undamaged leaf pair on an individual branch and located in direct sunlight.

### 2.3 Microsatellite genotyping and data quality

Leaves were dehydrated in silica gel, and genomic DNA was isolated from 20 mg of dry tissue with the DNeasy Plant Mini Kit (Qiagen) following the standard protocol, with an extended incubation step of 45 min. Initial tests included 17 previously‐published nuclear microsatellite loci (Cerón‐Souza, Bermingham, McMillan, & Jones, 2012; Cerón‐Souza, Rivera‐Ocasio, Funk, & McMillan, 2006; Mori, Zucchi, Sampaio, & Souza, 2010; Nettel, Rafii, & Dodd, 2005) (Appendix S2). Final tree genotypes included 12 of these loci combined into two multiplex reactions (Table S2). Polymerase chain reaction (PCR) conditions followed the PCR method for a single set of cycles with 35 cycles (as outlined in Culley et al., 2013), and we used the Type‐it Microsatellite PCR Kit (Qiagen). Total volume for each of two multiplex reactions was 6 μl with 2.5 μl Multiplex PCR Master Mix, 0.5 μl primer mix, 1 μl dH_2_O, and 2 μl (~20 ng) of genomic DNA (Table S2 details primer combinations and concentrations [μM] in each multiplex). PCR were performed on a T100 thermal cycler (Bio‐Rad). PCR products were separated on an ABI 96‐capillary 3730xl DNA Analyser with ROX size standard and scored in GeneMapper 5.1 (Applied Biosystems).

We evaluated potential genotyping errors in MICRO‐CHECKER 2.2.3 (van Oosterhout, Hutchinson, Wills, & Shipley, 2004) and estimated null allele frequencies with FreeNA (Chapuis & Estoup, 2007). We randomly reamplified and regenotyped 5% of DNA samples to assess genotyping accuracy and estimate a study error rate. We then tested for linkage disequilibrium and deviations from Hardy‐Weinberg equilibrium at each collection site after adjusting for multiple comparisons in FSTAT 2.9.3.2 (Goudet, 2002). POWSIM 4.1 (Ryman & Palm, 2006) was used to evaluate the resolving power of these microsatellite loci across all collection sites, and for each of the three distribution ranges separately.

### 2.4 USA *Avicennia germinans* neutral genetic diversity & structure

For each collection site, we calculated the number of polymorphic loci and private alleles in GenAlEx 6.5 (Peakall & Smouse, 2012). We calculated observed heterozygosity, unbiased gene diversity (H_S_), inbreeding coefficients, allelic richness (AR) standardized to minimum sample size (*n* = 9), and genetic differentiation (measured with *F*_ST_) with corresponding *p*‐values determined with 10^4^ permutations and adjusted for multiple comparisons in FSTAT 2.9.3.2. We also calculated G″_ST_ (Meirmans & Hedrick, 2011), D (Jost, 2008), and null‐allele‐corrected *F*_ST_ in FreeNA (Chapuis & Estoup, 2007) and these metrics were highly correlated with *F*_ST_ (Pearson's correlation, *r* = .96–1.0, *p* < .0001; Figure S2), so we present results only in terms of *F*_ST_. For all sites with *n* ≥ 23 genetic samples, we also calculated a more robust estimate of AR standardized to minimum sample size (*n* = 23) in FSTAT 2.9.3.2 for statistical analyses.

To assess variation among the three distribution ranges, we tested for differences in intrasite neutral genetic diversity (H_S_, AR; *n* = 12 per collection site) and intersite genetic differentiation within each distribution range (*F*_ST_; *n* = 6 per collection site in TX‐LA, *n* = 8 in WFL, *n* = 17 in EFL). *F*_ST_ sample sizes varied depending on the total number of collection sites with *n* ≥ 23 genetic samples for each distribution range. We used Kruskal‐Wallis tests and Dunn's tests for post hoc multiple comparisons with *p*‐values adjusted for the false discovery rate with the Benjamini‐Hochberg procedure. Unless otherwise noted, we performed statistical analyses in R 3.4.2 (R Core Team, 2013). We tested for a pattern of isolation by distance along each distribution range with Mantel tests of correlation between matrices of neutral genetic distances (intersite *F*_ST_/[1 − *F*_ST_] [Rousset, 1997]) and geographic distances (measured along the coastline between central points within each site in Google Earth 7.1.2.2041) in the R‐package ecodist (Goslee & Urban, 2007) with 10^4^ permutations to determine significance.

We visualized genetic structure across the entire USA range (*n* = 1,083 individuals) with STRUCTURE 2.3 (Pritchard, Stephens, & Donnelly, 2000) that determines the most likely number of population clusters (*K*) and assigns each sampled individual to these clusters based on multi‐locus genotypes. We used the admixture model with correlated allele frequencies, and did not consider geographic location. The analysis consisted of 10 replicate runs of 500,000 recorded steps after a burnin of 100,000 steps at each *K* value from 1 to 30. We used StrAuto (Chhatre & Emerson, 2017) in conjunction with GNU Parallel (Tange, 2011) to automate replicate runs across a 40‐core standalone computer. We used CLUMPAK (Kopelman, Mayzel, Jakobsson, Rosenberg, & Mayrose, 2015) with default settings to align replicate runs and visualize genetic structure at each *K* value.

Determining which *K* best fits a data set remains a debated topic. One method is to identify *K* with the greatest log probability [ln Pr(*X*|*K*)] or where values reach a relative plateau (Pritchard, Wen, & Falush, 2003). An alternative, the ∆*K* method, generally identifies the highest level of genetic structure, and may require subsequent analyses on data subsets to identify additional nested structure (Evanno, Regnaut, & Goudet, 2005). Use of both methods is recommended to better interpret patterns of genetic structure, while also considering species biology and including complementary analyses (Gilbert et al., 2012; Janes et al., 2017). We determined *K* with both ∆*K* and ln Pr(*X*|*K*) in STRUCTURE HARVESTER (Earl & VonHoldt, 2012). Based on our initial results, we performed subsequent analyses on data subsets from TX‐LA (*n* = 9 sites, 223 individuals) and Florida (*n* = 32 sites, 860 individuals), and then WFL (*n* = 9 sites, 270 individuals) and EFL (*n* = 18 sites, 540 individuals) separately. Run conditions were identical to the initial analysis, but we tested different ranges of *K* because of variation in collection site numbers (TX‐LA: *K* = 1–9; Florida: *K* = 1–30; WFL: *K* = 1–9; EFL: *K* = 1–18). We used the LOCPRIOR model (Hubisz, Falush, Stephens, & Pritchard, 2009) to assist the clustering analysis for TX‐LA only.

We performed a principal coordinates analysis (PCoA) with Nei's genetic distances in GenAlEx 6.5 as an additional line of evidence for population structure along the entire USA range. We then performed PCoA for each of the three distribution ranges separately. We plotted the first two axes with the R‐package ggplot2 (Wickham, 2011).

We tested explicit predictions of CMH (Table 1) along each of the three distribution ranges with Spearman's rank correlations between neutral genetic diversity (unbiased gene diversity, H_S_; allelic richness, AR) and latitude, and between genetic differentiation (*F*_ST_) and latitude. For each distribution range, latitude was highly correlated with distance to range core (measured as the distance along the coast from the most southern collection site), a recommended predictor variable for these analyses (Eckert et al., 2008) (TX‐LA: Pearson's correlation, *r* = .85, *p* < .0001; WFL: *r* = .96, *p* < .0001; EFL: *r* = 1.0, *p* < .0001). Use of either predictor did not qualitatively change correlation results.

Rare alleles can spread and become more frequent at expanding range margins because of strong genetic drift, a process called allele surfing (Excoffier & Ray, 2008 and citations within). We tested for this pattern of genetic drift at each of these three currently‐expanding range margins, with a modification of the method outlined in Griffin and Willi (2014). We first identified the most common alleles at each microsatellite loci within each of the three range cores. Most loci exhibited 1–2 predominant allele(s) within each collection site. These alleles were present across range core sites at a frequency of 0.95 ± 0.07 (standard deviation; *SD*) in TX‐LA, 0.91 ± 0.07 in WFL, and 0.93 ± 0.08 in EFL, and included at least 75% of the total allele pool per locus. We discarded these common alleles and filtered the remaining alleles based on the following criteria: (a) present in range margin site(s), (b) present in ≥ 2 range core sites, and (c) at least three copies (5% of collection site) present at range margin site(s). We included the last two criteria to avoid extremely rare or private alleles from skewing results. The resulting data set consisted of nine alleles in TX‐LA (18% of TX‐LA alleles), eight in WFL (11% of WFL alleles), and eight in EFL (11% of EFL alleles), with frequencies across range core sites of 0.06 ± 0.03 (*SD*), 0.09 ± 0.04, and 0.07 ± 0.04, respectively. We calculated the ratio of each allele's mean range margin frequency to its mean range core frequency, and transformed with the natural logarithm. We used a one‐sided *t *test to determine whether the ratio of margin to core allele frequency for each of the three range margins was greater than zero.

### 2.5 Leaf functional traits

We measured five leaf functional traits: area, length, width, ratio length:width, and specific leaf area. Area (cm^2^) was measured with an area meter (Model 2100, LI‐COR Inc.). Length (cm) was measured from the leaf tip to the start of the petiole and width (cm) was measured at the widest point of the leaf. Ratio length:width was also calculated as this trait proved informative to differentiate populations of *A. marina*, another member of the same genus (Saenger & Brooks, 2008). We dried leaves at 60°C for 48 hr until constant weight and measured dry weights (g). Specific leaf area (cm^2^/g) was measured as leaf area divided by dry weight. We measured these traits for 10 leaves per tree and used the mean value for each tree for analyses. For each of the three distribution ranges separately, we used principal components analysis (PCA) to reduce these functional traits into a limited number of uncorrelated variables. We log‐transformed trait data, centred and scaled values (mean = 0, variance = 1), and performed PCA with the R‐function *prcomp*. We retained principal components (PC) with eigenvalues >1.

We tested an implicit prediction of CMH (Table 1) by evaluating whether range‐margin *A. germinans* exhibit functional trait variation better suited to tolerate cold stress compared to range‐core conspecifics. We performed Spearman's rank correlations between functional trait PC and latitude along each of the three distribution ranges. Microsatellite genotype and functional trait data are available at the Dryad digital repository (Kennedy, Preziosi, Rowntree, & Feller, 2020).

## 3 RESULTS

### 3.1 Microsatellite data quality

Across the 12 nuclear microsatellite loci, potential null alleles were identified at 15% (75 of 492) of collection site – microsatellite locus pairs, but at low frequency (0.05 ± 0.07 [*SD*]) (Tables S3 and S4). The estimated error rate was also low at 1.39% (14 errors out of 1,007 allele comparisons), and we removed these locus‐specific errors from the data set. We found no evidence of linkage disequilibrium and only 2% (12 of 492) of collection site – microsatellite locus pairs deviated from Hardy‐Weinberg equilibrium. POWSIM results indicated that a true *F*_ST_ ≥ 0.005 could be detected with 100% probability across all collection sites, presumably more than sufficient resolution based on observed population structure (overall *F*_ST_ = 0.35). Resolution remained high for subsets from West Florida (WFL) and East Florida (EFL) (true *F*_ST_ ≥ 0.005 detected with 95.1% and 99.9% probability, respectively), but with a marked decrease for Texas‐Louisiana (TX‐LA) (detected with 71.1% probability).

### 3.2 USA *Avicennia germinans* neutral genetic diversity & structure

We found a total of 95 alleles among 1,083 individuals. All 12 microsatellite loci were polymorphic within the most southern collection sites in Florida (except for three sites with more limited sampling, *n* = 10 per site) and had increased monomorphism towards range margins (Table S1). In contrast, multiple loci were monomorphic across TX‐LA (maximum polymorphism = 8 of 12 loci per site). Twenty private alleles were identified at low frequencies (0.02 ± 0.01 [*SD*]) and were found only within range‐core sites in Florida, but within both core and margin sites in TX‐LA. Neutral genetic diversity was highest at lower latitudes in Florida, with maximum values in the southeast, and lowest at Florida range margins and across TX‐LA (Table S1; Figure 1d). Significant inbreeding (*F*_IS_) was detected within multiple sites, but at higher frequency across range margins (seven of 10 sites) compared to range cores (12 of 31 sites). We found significant genetic differentiation across all collection sites (*F*_ST_ = 0.35, *p* < .0001) with a range of intersite values from –0.02 to 0.77 (Table S5).

Among the three distribution ranges, both measures of neutral genetic diversity were significantly higher in WFL and EFL compared to TX‐LA (unbiased gene diversity, H_S_: Kruskal‐Wallis chi‐squared, H(2) = 61.0, *p* < .0001; post hoc tests, *p* < .0001, *p* < .0001) (allelic richness, AR: H(2) = 35.3, *p* < .0001; post hoc tests, *p* < .0001, *p* < .0001), but values were not significantly different between WFL and EFL (H_S_: post hoc tests, *p* = .07; AR: post hoc tests, *p* = .29) (Figures S3a,b). In contrast, genetic differentiation (*F*_ST_) was significantly lower in TX‐LA compared to WFL and EFL (H[2] = 67.1, *p* < .0001; post hoc tests, *p* < .0001, *p* < .0001) and significantly lower in EFL compared to WFL (post hoc tests, *p* = .01) (Figure S3c). We found evidence of isolation by distance along all three distribution ranges, with the highest correlation between neutral genetic distances and geographic distances along WFL (*r*_M_ = .85, *p* = .0003) compared to EFL (*r*_M_ = .33, *p* = .021) and TX‐LA (*r*_M_ = .54, *p* = .034).

Consistent with findings from Janes et al. (2017), ∆*K* identified *K* = 2 across all STRUCTURE analyses (except for the TX‐LA subset); whereas, ln Pr(*X*|*K*) identified additional levels of genetic structure that coincided with geographic location. We interpreted these differences between methods as the highest level of genetic structure (∆*K*) and finer‐scale genetic structure [ln Pr(*X*|*K*)] for each analysis (Appendix S3, Figures S4–S10 provide detailed explanations of *K* choice, STRUCTURE results, and PCoA results). Across the USA range, ∆*K* identified a clear separation between TX‐LA and Florida (Figure S5) and ln Pr(*X*|*K*) identified *K* = 11, with additional delineations between both WFL and EFL range margins and their respective cores, and admixture along multiple sections of the Florida range core (Figure 1e). Analysis of the Florida subset reached the same conclusions as the entire USA range (Figure S6). For WFL, ∆*K* identified a separation between range core and margin (Figure S7) and ln Pr(*X*|*K*) identified *K* = 5, with intersite admixture within the range core and sharp delineations at the most northern margin sites (Figure 1f). For EFL, results were analogous to WFL (∆*K* = 2, Figure S8; ln Pr(*X*|*K*) identified *K* = 7), except for an anomalous example of within‐range‐core delineation (site code: PI) (Figure 1f). For TX‐LA, we utilized the LOCPRIOR model and both ∆*K* and ln Pr(*X*|*K*) identified *K* = 4, with separation into southern, central, and northern clusters, plus a seemingly noninformative fourth cluster across all sites (Figure S9). *K* = 3 identified only the biologically‐sensible clusters, with admixture at a recently‐recolonized range‐margin site (code: TP) (Figure 1f). PCoA was consistent with STRUCTURE, but indicated further separation between northern Texas and Louisiana (Figure S10).

**Figure 2:**
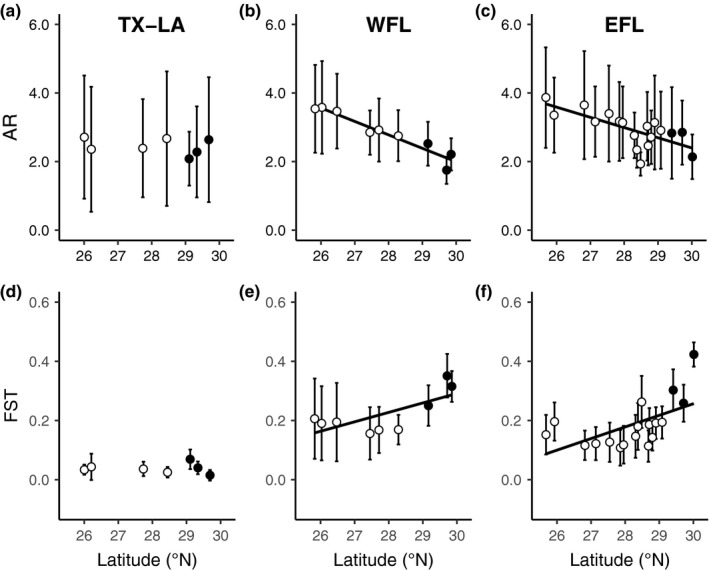
Changes in neutral genetic diversity (allelic richness; AR) and differentiation (fixation index; FST) along (a, d) Texas‐Louisiana (TX‐LA), (b, e) West Florida (WFL), and (c, f) East Florida (EFL). Significant (*p* < .05) correlations are depicted with a solid line. Range core sites are shown in white and margin sites in black. Error bars indicate 95% confidence intervals. AR; *n* = 12 per collection site. *F*_ST_; *n* = 6 per collection site in TX‐LA, *n* = 8 in WFL, *n* = 17 in EFL

We found that EFL and WFL distribution ranges conformed to CMH predictions, but TX‐LA did not (Table 1; Figure 2). In EFL, neutral genetic diversity (unbiased gene diversity, H_S_; allelic richness, AR) was negatively correlated with latitude (Spearman's rank correlation coefficient, *r*_s_ = –.20, *p* = .004; *r*_s_ = –.22, *p* = .001, respectively) and genetic differentiation (*F*_ST_) was positively correlated with latitude (*r*_s_ = .38, *p* < .0001). In WFL, H_S_ was not correlated with latitude (*r*_s_ = –.02, *p* = .83), whereas AR was negatively correlated with latitude (*r*_s_ = –.32, *p* = .0007) and *F*_ST_ was positively correlated with latitude (*r*_s_ = .36, *p* = .002). In TX‐LA, neither H_S_ or AR were correlated with latitude (*r*_s_ = .05, *p* = .65; *r*_s_ = .04, *p* = .74, respectively) and *F*_ST_ was not correlated with latitude (*r*_s_ = –.08, *p* = .59).

We found evidence of genetic drift at each of the three range margins (Table 2). Rare alleles in the range core were more frequent in the range margin in EFL (ln margin:core allele frequencies; one‐sided *t *test, *t*[7] = 3.40, *p* = .006), WFL (*t*[7] = 4.08, *p* = .002), and TX‐LA (*t*[8] = 3.74, *p* = .003). WFL exhibited the highest mean increase in rare allele frequencies (4.85 ± 3.12 [*SD*] times greater than range core) compared to EFL (2.66 ± 1.48) and TX‐LA (2.73 ± 2.28).

**Table 2:** Evidence of genetic drift at the expanding Texas‐Louisiana (TX‐LA), West Florida (WFL), and East Florida (EFL) range margins

Distribution	Location	Sites	Total alleles	% allele decrease	Rare alleles	Margin:core	ln (margin:core)	*t*	*df*	*p*‐value
TX‐LA	Core	5	45	0.16	9	2.73 ± 2.28	0.80 ± 0.64	3.74	8	.003
	Margin	3	38							
WFL	Core	6	67	0.45	8	4.85 ± 3.12	1.31 ± 0.91	4.08	7	.002
	Margin	3	37							
EFL	Core	15	71	0.44	8	2.66 ± 1.48	0.81 ± 0.67	3.40	7	.006
	Margin	3	40							

Note

Sites, number of collection sites; total alleles, number of alleles found within each location; % allele decrease, percent decrease in number of alleles from range‐core to margin; rare alleles, number of identified range‐core rare alleles used in analysis. Error indicates 95% confidence intervals.

### 3.3 Leaf functional traits

**Figure 3:**
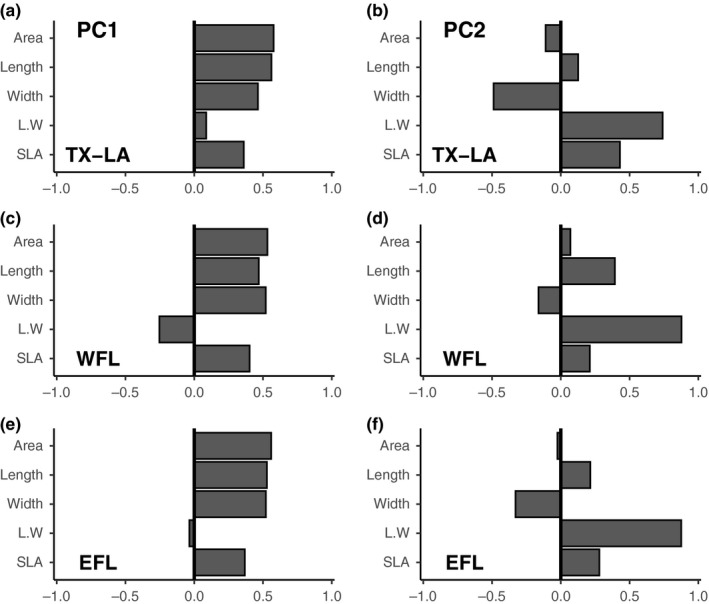
Loadings on principal components (PC) of variation in functional traits of leaves for Texas‐Louisiana (TX‐LA), West Florida (WFL), and East Florida (EFL). Left: PC1 for TX‐LA, WFL, and EFL (a, c, e), which accounted for 55.4%, 69.1%, and 62.1% of the total variation, respectively. Right: PC2 for TX‐LA, WFL, and EFL (b, d, f), which accounted for 32.1%, 20.1%, and 24.9% of the total variation, respectively. L.W, ratio length:width; SLA, specific leaf area

We found the largest leaves and highest specific leaf area (SLA) in Southwest and Southeast Florida; whereas, we found lower values towards Florida range margins and across all TX‐LA sites (Table S1). PCA of functional traits resulted in similar patterns for each of the three distribution ranges (Figure 3). The first two principal components (PC) had eigenvalues >1 for each distribution range, and accounted for 87% (62.1% and 24.9%, respectively) of the total variation in EFL, 89.3% (69.1% and 20.1%, respectively) in WFL, and 87.5% (55.4% and 32.1%, respectively) in TX‐LA.

Leaf size (area, length and width) and life history strategy (SLA) traits had strong, positive loadings on PC1 (Figure 3). Negative values along PC1 are indicative of smaller leaves and lower SLA, both traits consistent with cold tolerance. Leaf shape (ratio length:width) had strong, positive loadings on PC2, reinforced by negative loadings for width in EFL and TX‐LA and positive loadings for length in WFL (Figure 3). Positive values along PC2 are indicative of longer, narrower leaves, a trait associated with greater light capture efficiency (Takenaka, 1994), not with cold tolerance. SLA had comparatively higher positive loadings on PC2 for TX‐LA compared to Florida distribution ranges, so we interpreted larger PC2 values in TX‐LA as indicative of longer, narrower leaves with higher SLA.

**Figure 4:**
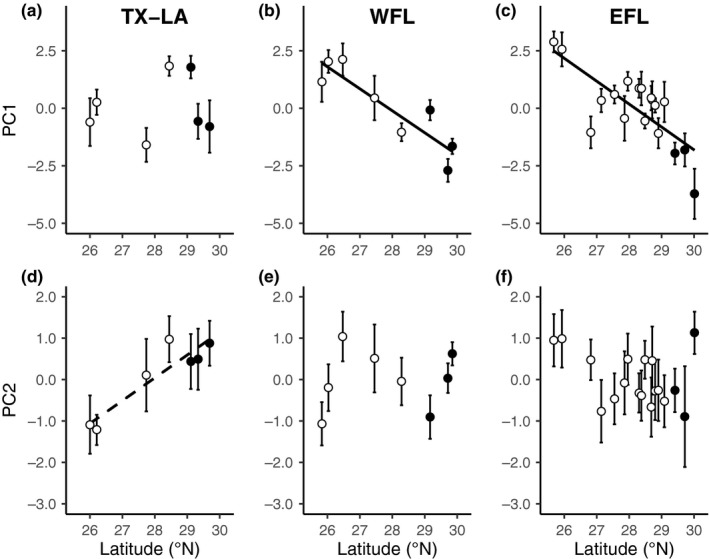
Changes in principal components (PC1, PC2) of variation in functional traits of leaves along (a, d) Texas‐Louisiana (TX‐LA), (b, e) West Florida (WFL), and (c, f) East Florida (EFL). Significant (*p* < .05) correlations are depicted with a solid line, and marginal nonsignificant (*p* = .052) correlations are depicted with a dashed line. Range core sites are shown in white and margin sites in black. Error bars indicate 95% confidence intervals

In contrast to theory, EFL and WFL range margins exhibited functional traits consistent with greater cold tolerance, and all TX‐LA collection sites exhibited similar cold‐tolerant traits (Table 1). PC1 was negatively correlated with latitude along EFL (*r*_s_ = –.69, *p* = .002) and WFL (*r*_s_ = –.86, *p* = .007), indicative of functional trait variation better suited to tolerate cold stress at these range margins, but PC1 was not correlated with latitude along TX‐LA (*r*_s_ = 0, *p* = .99) (Figure 4). PC2 was not significantly correlated with latitude along EFL (*r*_s_ = –.24, *p* = .34) or WFL (*r*_s_ = .38, *p* = .35), but was positively correlated (albeit marginally nonsignificant) with latitude along TX‐LA (*r*_s_ = .75, *p* = .052) (Figure 4).

## 4 DISCUSSION

We still lack a clear understanding of what controls distributional limits of species (Parmesan et al., 2005), in part because of limited empirical data across large spatial scales (Abeli et al., 2014; Sagarin et al., 2006). Here, we provide insights into the lack of consensus regarding the central‐marginal hypothesis (CMH) and also into the implications of this theory. The model system we used naturally exhibits parallel gradients in latitude, ecological marginality, and postglacial recolonization, which alleviates many confounding factors that may hinder research, and also provides replicate distribution ranges that either conform or do not conform to the underlying “abundant‐centre” assumptions of CMH (i.e., decline in ecological conditions and abundances towards range margins). We demonstrated that CMH is validated only when “abundant‐centre” assumptions are met, and that reduced neutral genetic variation at range margins does not constrain shifts in functional trait variation along climatic gradients.

### 4.1 Explicit CMH predictions

A lack of consensus about the predictions of CMH is thought to be the product of multiple confounding factors and the fact that the underlying “abundant‐centre” assumptions of this theory are often not met (Eckert et al., 2008; Pironon et al., 2017). Our study echoes this sentiment as we found support for CMH, but with the caveat that distribution ranges must meet these assumptions. Therefore, analogous to assumption testing in statistics, research into genetic changes across species' distributions should first confirm whether “abundant‐centre” assumptions are met. For example, ecological niche modelling has proven successful in identifying patterns in ecological gradients across core to margin transects (Lira‐Noriega & Manthey, 2014; Micheletti & Storfer, 2015; Pironon et al., 2015).

We contend that mangroves simplify this process with their easily‐defined distributions that generally exhibit reduced abundances as conditions become more marginal towards climate‐sensitive poleward range limits (Osland, Feher, et al., 2017). USA mangroves also provide three natural replicates of core to margin distribution ranges. Comparing variation across multiple distributions of the same species can provide greater insights into the processes shaping genetic change (Griffin & Willi, 2014; Kennedy et al., 2017; Leydet, Grupstra, Coma, Ribes, & Hellberg, 2018; Micheletti & Storfer, 2015). For instance, West Florida (WFL) and East Florida (EFL) exhibited a similar decline in neutral genetic diversity, with reductions in mean allelic richness of almost 50% from south to north, and greatest intersite differentiation at the northern range margins, consistent with latitudinal reductions in mangrove abundances along these coastlines (Osland, Feher, et al., 2017). However, we found a stronger effect of genetic drift at the WFL range margin, a pattern also observed in a co‐occurring mangrove species, *Rhizophora mangle* (Hodel, Souza Cortez, Soltis, & Soltis, 2016; Kennedy et al., 2017). Greater genetic drift at the WFL range margin may be explained by multiple factors, including greater geographic isolation from the range core, more extreme minimum annual temperatures, limited ocean‐current‐driven propagule dispersal (Kennedy et al., 2017), and restricted colonization due to propagule predation (Langston, Kaplan, & Angelini, 2017).

An anomaly within the EFL range core (site code: PI) suggests another potential caveat to CMH. This collection site exhibited strong within‐range‐core differentiation and lowest neutral genetic diversity along this distribution range. Land modifications associated with this site's conversion into a conservation area, plus limited hydrological exchange because this portion of the EFL lagoon system experiences highest water residence times (Smith, 1993), probably explain this anomalous pattern. Identifying effects of human activity on intraspecific genetic variation is a research priority (Guo, 2012), and this example highlights that deviations from CMH predictions may also be the product of anthropogenic changes and context‐specific environmental factors that may restrict recruitment to local sources.

We found a strong delineation between *A. germinans* in Florida and those in Texas‐Louisiana (TX‐LA), consistent with independent post‐Pleistocene recolonization routes (Sherrod & McMillan, 1985) and the potential role of the Mississippi River as a biogeographic barrier (Soltis, Morris, McLachlan, Manos, & Soltis, 2006). In contrast to Florida, TX‐LA did not conform to the underlying “abundant‐centre” assumptions and, as such, did not support CMH predictions. TX‐LA *A. germinans* are essentially a series of range margins because the entire distribution experiences marginal environmental conditions. Research from Atlantic Mexico, directly south of TX‐LA and closer to this species' true range core, found highest allelic richness at lower latitudes (18°N–20°N), with values analogous to our lower‐latitude Florida collection sites, and lowest allelic richness in northern Mexico (25.9°N; adjacent to our most southern site) (Ochoa‐Zavala, Jaramillo‐Correa, Piñero, Nettel‐Hernanz, & Núñez‐Farfán, 2019). Combining genetic coverage from Mexico into TX‐LA highlights two important points. First, reduced neutral genetic variation seems to be a characteristic of TX‐LA *A. germinans*, presumably the product of restricted population sizes, recurrent fluctuations in abundance during periods of increased aridity and/or cold, and geographical isolation among distributional centres and from more continuous forests in Mexico (Sherrod & McMillan, 1981). Second, species range margins are not always restricted to their geographical limits, and instead may exist across extensive spatial scales (~1,000 km in this case) because of widespread ecologically‐marginal conditions.

As mangroves generally conform to these “abundant‐centre” assumptions, we would expect additional support for CMH across their pantropical distribution. Osland, Feher, et al. (2017) evaluated mangrove distributions worldwide and determined that most range limits were controlled by either temperature or precipitation, with only four geographic regions influenced by both factors: Texas‐Louisiana (TX‐LA), Pacific Mexico, Western Australia, and the Middle East. CMH predictions are supported along multiple mangrove distributions controlled by either temperature or precipitation (Arnaud‐Haond et al., 2006; De Ryck et al., 2016; Francisco, Mori, Alves, Tambarussi, & de Souza, 2018; Kennedy et al., 2017; Maguire, Saenger, Baverstock, & Henry, 2000; Pil et al., 2011; Sugai et al., 2016), consistent with our findings along temperature‐controlled Florida. CMH is also supported in Pacific Mexico (Ochoa‐Zavala et al., 2019; Sandoval‐Castro et al., 2014, 2012) and Western Australia (Arnaud‐Haond et al., 2006; Binks et al., 2019), where parallel declines in temperature and precipitation limit mangrove distributions. Our finding that CMH is not supported across TX‐LA seems to be an exception to the general rule in mangroves, although the Middle East may also prove to be an exception as the entire region is precipitation limited (Osland, Feher, et al., 2017). Yet, CMH predictions are also not supported directly south of TX‐LA where Atlantic Mexican *A. germinans* did not exhibit a systematic decline in genetic diversity due to persistence in multiple glacial refugia (Ochoa‐Zavala et al., 2019), and in the wider Caribbean where post‐glacial expansion seemingly occurred along separate dispersal pathways (Kennedy et al., 2016). Mangroves, and coastal species in general (Sagarin et al., 2006), seem to provide ideal models to test many large‐scale ecological theories, but deviations may exist due to nonconformity to underlying “abundant‐centre” assumptions (as shown here in TX‐LA), and to confounding effects of variation in past distributional fluctuations (as previously shown in Atlantic Mexico and the wider Caribbean), which reiterates the need to incorporate assumption testing into future empirical research.

### 4.2 Implicit CMH prediction

The underlying importance of documenting genetic changes towards range margins is that limited adaptive genetic variation could reduce evolutionary potential and constrain adaptation to novel environmental conditions, a possible mechanism defining distributional limits (Bridle & Vines, 2007). We found that reduced neutral genetic variation at three range margins was not a constraint to shifts in functional trait variation consistent with a response to cold stress. Although USA range‐margin *A. germinans* are smaller than conspecifics towards the range core (Feher et al., 2017), these small‐statured individuals exhibited a change in functional traits consistent with greater cold tolerance. A similar trade‐off in plant size and leaf traits exists for *A. germinans* along Atlantic Mexico (Méndez‐Alonzo, López‐Portillo, & Rivera‐Monroy, 2008), and freeze experiments have demonstrated that this transition towards cold‐tolerant leaf traits in East Florida *A. germinans* correlates with greater freeze tolerance at the range margin (Cook‐Patton et al., 2015). Additional systematic changes towards USA mangrove range limits include narrower xylem vessel architecture (Madrid et al., 2014), precocious reproduction and increased propagule size (Dangremond & Feller, 2016), and greater reproductive success (Goldberg & Heine, 2017). Together, these observations are consistent with evidence to date that reduced neutral genetic variation at range margins does not necessarily diminish species performance (Abeli et al., 2014; Pironon et al., 2017), and add to our growing understanding of the importance of intraspecific trait variation in explaining ecological patterns (Siefert et al., 2015).

Functional trait variation of *A. germinans* leaves towards USA range margins mirrored gradients in climatic factors (i.e., temperature, precipitation) that control these distributional limits. Both Florida distribution ranges exhibited a change in leaf traits towards those better suited to tolerate cold, consistent with gradients in minimum winter temperatures. However, while WFL exhibited a more continuous change in leaf traits towards the range margin, EFL seemed to exhibit a more abrupt change, in particular at the most northern collection site. Trait variation along environmental gradients can vary depending on rates of gene flow and the strength of genetic drift (Polechová, 2018). Differences between WFL and EFL in these two factors (i.e., WFL: stronger pattern of isolation by distance, stronger effect of genetic drift at the range margin) may explain these patterns in trait variation. In contrast, functional traits across TX‐LA were comparable to those at Florida range margins, presumably the product of inverse gradients in temperature and precipitation that may blur geographic patterns, as similar leaf traits are consistent with both cold and drought tolerance (Knight & Ackerly, 2003). Our observation of a trend towards longer, narrower leaves with higher specific leaf area (SLA) at the TX‐LA northern range margin is also consistent with these inverse climatic gradients. A cumulative effect of both arid conditions and periodic freeze events could explain lowest SLA in southern sites (Poorter et al., 2009), with higher SLA as rainfall increases towards the higher‐latitude range margin. Less sunlight and greater abundance of co‐occurring salt marsh at higher latitude could then explain changes in leaf shape as light capture becomes more critical (Takenaka, 1994).

Observations of trait variation towards range margins seldom address the relative contributions of genetic differences and environmentally‐induced trait plasticity in explaining these patterns (Chuang & Peterson, 2016). Our measurements of leaf traits in situ and of putative neutral genetic variation with microsatellite loci also cannot address this question. Instead, common garden and reciprocal transplant experiments are needed to achieve a conclusive understanding of the mechanisms shaping functional trait variation at these range margins. Common garden experiments with *A. germinans* found greater chill tolerance in offspring from Texas compared to more‐equatorial regions (Markley, McMillan, & Thompson, 1982), and an over‐the‐edge transplant experiment (i.e., individuals transplanted beyond current range limits) demonstrated greater post‐freeze survival in seedlings from sources where freezes are common (Hayes et al., 2020). However, mangroves also exhibit high levels of trait plasticity in response to environmental cues (Feller et al., 2010). Additional over‐the‐edge transplant experiments will also further our understanding of whether these range margins are ecological niche limits or the product of dispersal limitation, important insight to better predict responses to climate change (Lee‐Yaw et al., 2016).

Considering ongoing, rapid expansion at all USA range margins (Rogers & Krauss, 2018) and further expansion forecast with climate change (Cavanaugh et al., 2015; Osland, Day, et al., 2017), plus the fact that range margins are probably the primary source of recruits beyond distributional limits (Hampe, 2011), USA range‐margin *A. germinans* appear well‐equipped to thrive in their marginal environment, unless directly impacted by anthropogenic changes. This continued proliferation will result in wide‐reaching community‐level effects (Diskin & Smee, 2017; Guo et al., 2017; Kelleway et al., 2017).

In conclusion, model systems that meet underlying assumptions and alleviate the influence of common confounding factors can provide important insights into many large‐scale ecological theories (Sagarin et al., 2006). We utilized a widespread mangrove species that naturally controls for common confounding factors to demonstrate that the central‐marginal hypothesis (CMH) is validated, but only when underlying “abundant‐centre” assumptions are met, and that reduced neutral genetic variation at range margins does not constrain shifts in functional trait variation along climatic gradients. Considering that many species do not conform to “abundant‐centre” assumptions (Sagarin & Gaines, 2002; Santini et al., 2019) and that numerous confounding factors can influence genetic patterns (Eckert et al., 2008), our findings support previous accounts that CMH does not represent a general rule across species (Pironon et al., 2017), with deviations from CMH probably becoming more common with climate change and greater anthropogenic pressures that can reduce and fragment suitable habitat. Finally, we agree with the framework proposed by Pironon et al. (2017) that research needs to employ an integrated approach that not only considers geographic gradients, but also ecological and historical gradients, when interpreting patterns of genetic and trait variation across broad spatial scales.

## AUTHOR CONTRIBUTIONS

J.P.K., and I.C.F. designed the research. J.P.K. performed the research and analyzed the data. R.F.P., J.K.R., and I.C.F. supervised the research. J.P.K. wrote the manuscript with input from all coauthors.

## Data Availability

Microsatellite genotype data and functional trait data are publicly available on Dryad: https://doi.org/10.5061/dryad.69p8cz8xh
