# Supplementary material for: Is the central‐marginal hypothesis a general rule? Evidence from three distributions of an expanding mangrove species, Avicennia germinans (L.) L
Source: Mol Ecol. 2020 Feb 14;29(4):704–19. doi: 10.1111/mec.15365 (PMC7065085; doi:10.1111/mec.15365)
Supplement: Supplementary file 1 [file MEC-29-704-s001.pdf]

MOLECULAR ECOLOGY

**Supplemental Information for:**

# Is the central-marginal hypothesis a general rule? Evidence from three distributions of an expanding mangrove species, *Avicennia germinans* (L.) L.

John Paul Kennedy[1,2], Richard F. Preziosi[2], Jennifer K. Rowntree[2] and Ilka C. Feller[3]
[1]Smithsonian Marine Station, Smithsonian Institution, Fort Pierce, Florida, USA
[2]Ecology and Environment Research Centre, Department of Natural Sciences, Faculty of Science and Engineering, Manchester Metropolitan University, Manchester, UK
[3]Smithsonian Environmental Research Center, Smithsonian Institution, Edgewater, Maryland, USA

## Table of Contents:


| | |
|---|---|
| **Figure S1** | Page 2 |
| **Appendix S1** | Page 3 |
| **Appendix S2** | Page 4 |
| **Figure S2** | Page 5 |
| **Figure S3** | Page 6 |
| **Appendix S3** | Page 7-9 |
| **Figure S4** | Page 10 |
| **Figures S5 – S9** | Page 11-12 |
| **Figure S10** | Page 13 |

MOLECULAR ECOLOGY

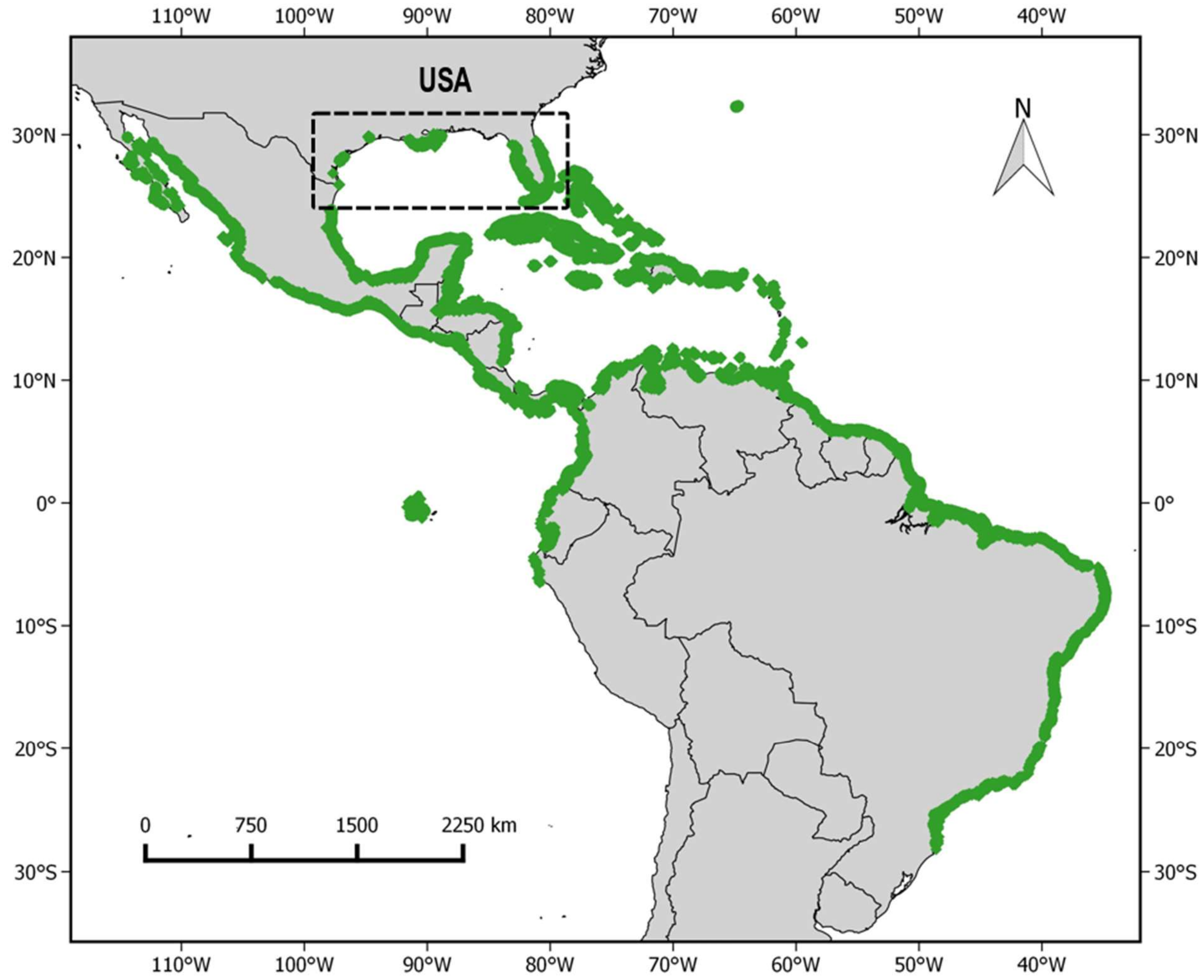


Figure S1. Distribution range of neotropical mangroves (Giri et al., 2011). USA mangroves provide extensions of the more-equatorial range core that eventually transition into climate-sensitive northern range margins along three discrete coastlines (Texas-Louisiana, West Florida, and East Florida).

MOLECULAR ECOLOGY

**Appendix S1: Latitudinal gradients in climatic factors along Texas-Louisiana, West Florida, and East Florida**

Mangrove distributional limits in the USA are controlled by latitudinal gradients in minimum temperatures along West Florida (WFL) and East Florida (EFL), and inverse latitudinal gradients in minimum temperatures and precipitation along Texas-Louisiana (TX-LA) (Cavanaugh et al., 2018; Osland et al., 2017). To characterize these gradients, we used datasets from 1980-2017 of mean minimum annual temperature and mean annual precipitation, with a 0.5 degree x 0.5 degree resolution (Figure 1b, c in the main text), available from the NOAA CPC precipitation and temperature datasets (https://www.esrl.noaa.gov/psd/data/gridded/). We extracted values for each of our TX-LA, WFL, and EFL collection sites with the 'point sampling tool' in QGIS 2.8.3 (QGIS Development Team, 2017). Consistent with previous research, minimum temperatures exhibited strong negative correlations with latitude along all three distribution ranges: TX-LA (Pearson's correlation, $r = -0.84$, $p = 0.02$), WFL ($r = -0.96$, $p = 0.0002$), and EFL ($r = -0.97$, $p < 0.0001$). Changes in mean minimum temperature from south to north along these distribution ranges were more extreme in WFL (2.6 to -6.9 ºC) and EFL (4.2 to -3.6 ºC) compared to TX-LA (-0.2 to -3.7 ºC), with the most southern collection sites in TX-LA exhibiting colder minimum temperatures than in WFL and EFL. Mean annual precipitation exhibited a strong positive correlation with latitude along TX-LA ($r = 0.89$, $p = 0.007$), with less than half the amount of rainfall at the southern range core compared to the northern range margin (701.5 to 1568.0 mm).

**Appendix S2: Microsatellite testing**

Trees were initially genotyped at 17 nuclear microsatellite loci: CTT_001, CA_002, CT_003 (Cerón-Souza, Rivera-Ocasio, Funk, & McMillan, 2006), GT_006, CT_004, CAT_004 (Cerón-Souza, Bermingham, McMillan, & Jones, 2012), Agerm1-02, Agerm1-07, Agerm1-11, Agerm1-12, Agerm1-14, Agerm1-15, Agerm1-16, Agerm1-18 (Mori, Zucchi, Sampaio, & Souza, 2010), AgD13, AgT4, and AgT8 (Nettel, Rafii, & Dodd, 2005). Three loci did not amplify consistently (Agerm1-02, Agerm1-07, Agerm1-15) and were discarded. Locus Agerm1-11 was discarded due to monomorphism across all samples. Agerm1-12 was discarded because MICRO-CHECKER 2.2.3 (van Oosterhout et al., 2004) identified potential null alleles in 68% (28 of 41) of collection sites. Final tree genotypes included 12 nuclear microsatellite loci combined into two multiplex reactions (Supporting information Table S2).

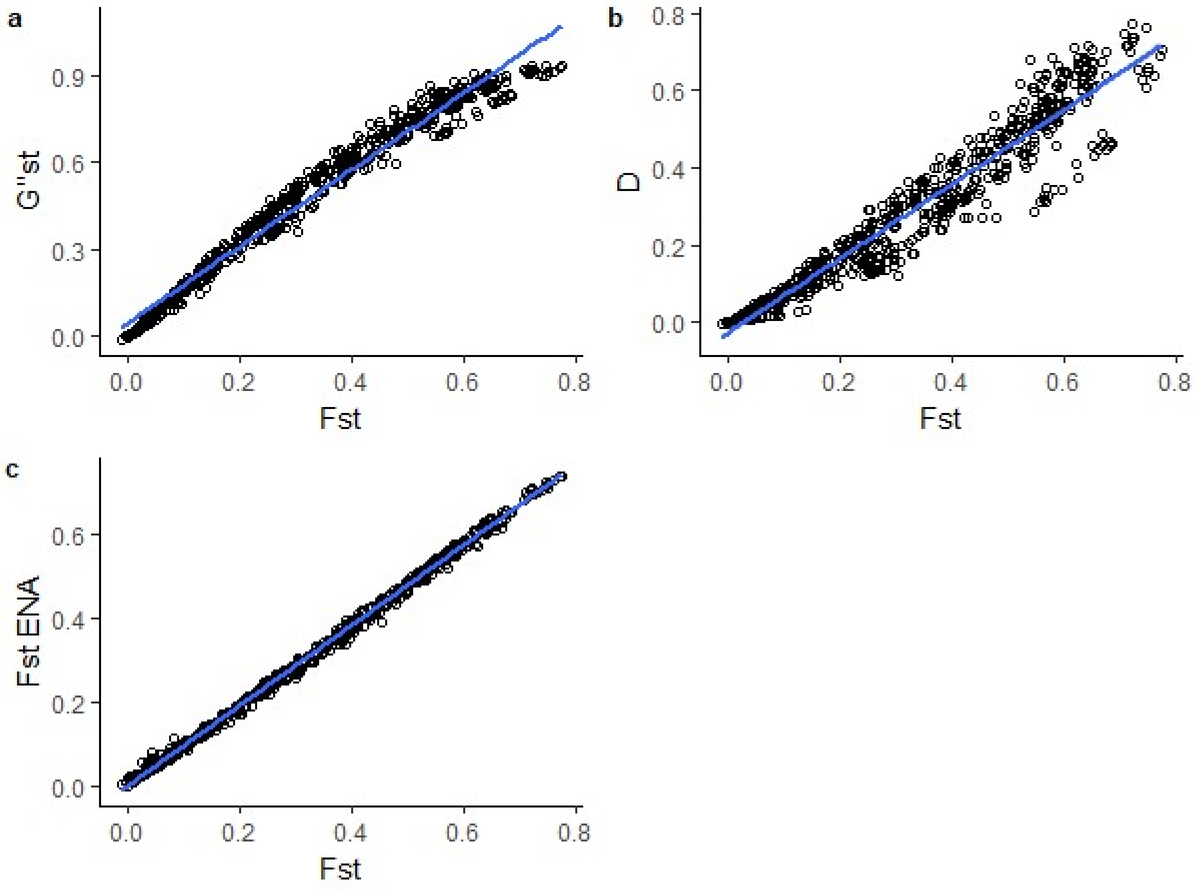


Figure S2. Comparison of $F_{ST}$ to three additional measures of genetic differentiation: (a) $G''_{ST}$ (Meirmans & Hedrick, 2011), (b) D (Jost, 2008) and (c) null-allele-corrected $F_{ST}$ calculated with FreeNA (Chapuis & Estoup, 2007). All three measures were highly correlated with FST (r = 0.988, 0.963, 0.999, respectively; $p < 0.0001$) as depicted with a solid blue line.

MOLECULAR ECOLOGY

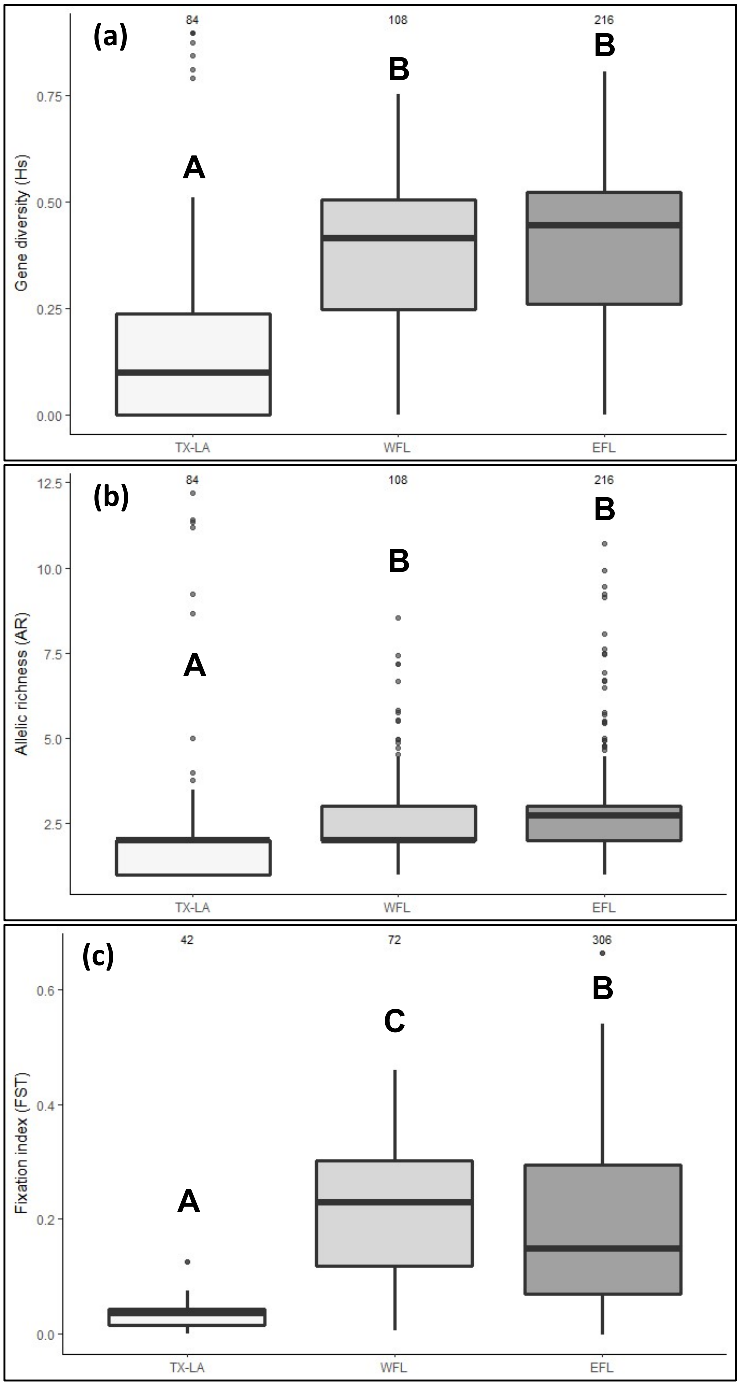


Figure S3. Boxplots of (a, b) intra-site genetic diversity and (c) inter-site genetic differentiation for each of the three distribution ranges from Texas-Louisiana (TX-LA), West Florida (WFL), and East Florida (EFL) (from left to right). Sample sizes shown at the top of each figure. Different letters indicate significant differences among distribution ranges ($p < 0.05$).

MOLECULAR ECOLOGY

**Appendix S3: Genetic structure analyses**

Consistent with findings from Janes et al. (2017), ΔK identified K = 2 across all STRUCTURE analyses (except for the TX-LA subset); whereas, ln Pr(X|K) identified additional levels of genetic structure. We interpreted these differences between methods as the highest level of genetic structure (ΔK) and finer-scale genetic structure [ln Pr(X|K)] for each analysis. ΔK identified K = 2 as the best fit across the entire USA distributional range, with TX-LA sites exclusively in the first cluster and Florida sites in the second, and varying degrees of admixture between both clusters for the most northern WFL and EFL sites (Supporting information Figures S4a and S5). ln Pr(X|K) increased greatly from K = 1 to 2 and then gradually until a relative plateau at K = 11 (Supporting information Figure S4a). From K = 2 to 11, additional well-defined clusters continued to be identified; whereas, K ≥ 12 identified low membership clusters across large geographic areas, consistent with a lack of further population structure (Pritchard, Wen, & Falush, 2003). K = 11 coincides with geographic location along the coastline, identifies a clear delineation between TX-LA and Florida, identifies clear delineations between both WFL and EFL range margins and their respective cores, and demonstrates substantial admixture along multiple portions of the Florida range core (Figure 1e in the main text).

Subsequent analysis of the Florida subset reached the same conclusions as the entire US distributional range analysis (Supporting information Figures S4b and S6). ΔK identified K = 2 as the best fit across Florida, with South Florida sites in the first cluster, Northeast Florida sites in the second, and admixture between both in Northwest Florida. ln Pr(X|K) increased until a relative plateau at K = 10. As in the initial analysis, there was a clear delineation between both WFL and EFL range margins and their respective cores and substantial admixture along larger portions of the range core. Although not a hard boundary, Southwest and Southeast Florida formed distinct clusters with a transition along the South Everglades and Florida Keys. Thus, subsequent analyses of the WFL and EFL subsets consisted of samples from Southwest Florida to the WFL range margin and from Southeast Florida to the EFL range margin, respectively. We excluded samples from South Florida (i.e., South Everglades and Florida Keys) because of their admixed assignments with both coastlines and because this area was not the focus of the current study.

MOLECULAR ECOLOGY

ΔK identified K = 2 as the best fit in WFL and demonstrated a separation between range core and margin, with admixture between the two clusters at their transition (Supporting information Figures S4c and S7). ln Pr(X|K) increased until a relative plateau at K = 5 (Supporting information Figure S4c), an identical result to both of the previous STRUCTURE analyses. K = 5 showed a gradual transition from southern to central sites, a similar transition to the range margin, and sharp delineations for the most northern margin sites (Figure 1f in the main text). Similar to WFL, ΔK identified K = 2 as the best fit in EFL and demonstrated a separation between range core and margin, with admixture between the two at their transition (Supporting information Figures S4d and S8). ln(Pr(X|K) increased until a relative plateau at K = 7 (Supporting information Figure S4d), with gradual transitions northward between clusters throughout the range core (except for an anomalous example of within range core delineation; site code: PI), a transition from the most northern core sites to the clearly delineated range margin, and a sharp delineation for the most northern margin site (Figure 1f in the main text).

Subsequent analysis of the TX-LA subset indicated a lack of population structure (Supporting information Figures S4e and S9a), so we utilized the LOCPRIOR model to assist the analysis and tested a slightly wider range of K values (K = 1-12). Both ΔK and ln Pr(X|K) identified K = 4 as the best fit (Supporting information Figure S4f). K = 4 coincides with geographical location along the coastline with southern, central, and northern clusters identified, plus a seemingly non-informative fourth cluster that covers the entire distribution range (Supporting information Figure S9b). K = 3 identifies only these three biologically sensible clusters, with unexpected admixture at a range margin site (Texas Point; code: TP) (Figure 1f in the main text). Complete mangrove die-back has been documented at Texas Point, with *A. germinans* present in 1954, not present in 1979 (Sherrod & McMillan, 1981) and only five trees identified in 2010 (Guo, Zhang, Lan, & Pennings, 2013).

PCoA for the entire USA range was consistent with STRUCTURE results, with a clear separation between TX-LA and Florida, the Florida range core clustered around the same dimensional space, and WFL and EFL range margins separated from their respective range cores (Supporting information Figure S10a). WFL and EFL PCoA were also consistent with STRUCTURE results, with a south to north gradient along the dimensional space and greatest

separation among sites towards the range margin (Supporting information Figures S10c and S10d, Supporting information). TX-LA PCoA was mostly consistent with STRUCTURE results, with separation among southern, central, and northern groups, and a lack of clear separation for one range margin site (code: TP). However, range margin sites that had previously been grouped together (code: GV, PF) are clearly distinct from each other and the other sites, an indication of separation between northern Texas and Louisiana (Supporting information Figure S10b).

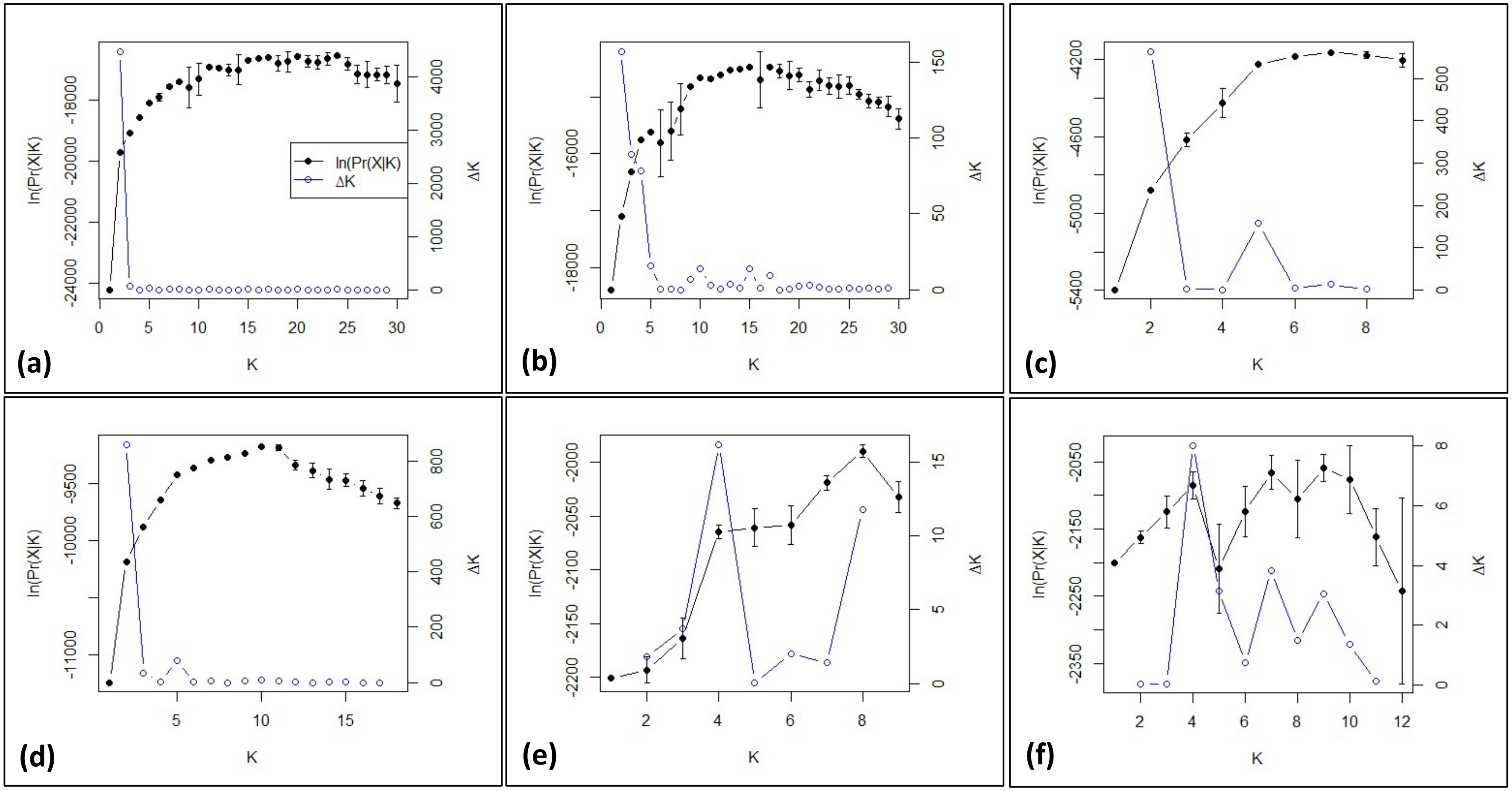


Figure S4. Plots of ΔK and ln Pr(X|K) from STRUCTURE analyses for (a) the entire USA distributional range, and subsets from (b) Florida, (c) West Florida, (d) East Florida, (e) Texas-Louisiana without LOCPRIOR model, and (f) Texas-Louisiana with LOCPRIOR.

MOLECULAR ECOLOGY

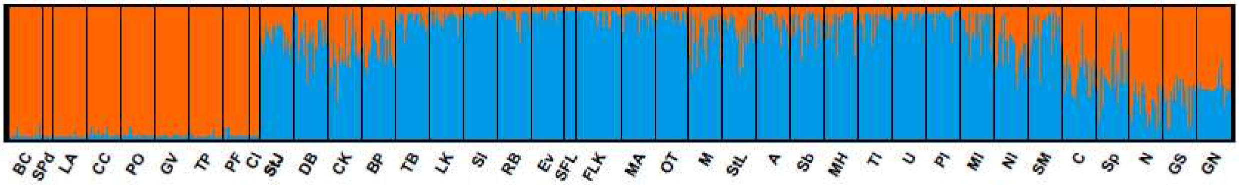


Figure S5. STRUCTURE results from the entire USA distributional range based on the ΔK method (identified K = 2). Refer to Supporting information Table S1 for site codes.

(a)

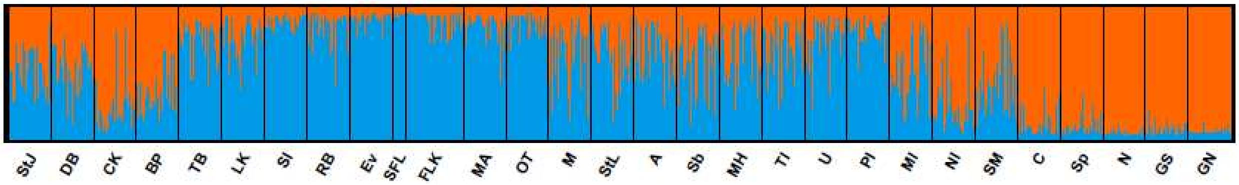


(b)

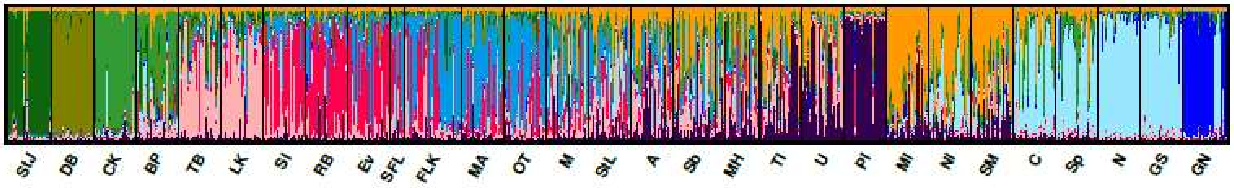


Figure S6. STRUCTURE results from Florida subset. (a) ΔK method identified K = 2; (b) ln Pr(X|K) increased until a relative plateau at K = 10. Note in panel b that Southwest and Southeast Florida formed distinct clusters (red and blue, respectively) with a transition along the South Everglades (site code: SFL) and Florida Keys (site code: FLK). Refer to Supporting information Table S1 for site codes.

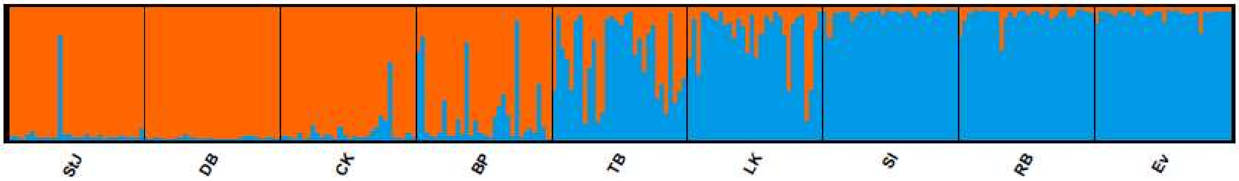


Figure S7. STRUCTURE results from West Florida subset. ΔK method identified K = 2. Refer to Supporting information Table S1 for site codes.

MOLECULAR ECOLOGY

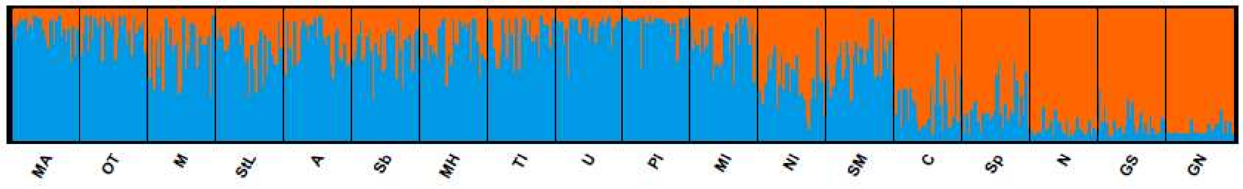


Figure S8. STRUCTURE results from East Florida subset. ΔK method identified K = 2. Refer to Supporting information Table S1 for site codes.

(a)

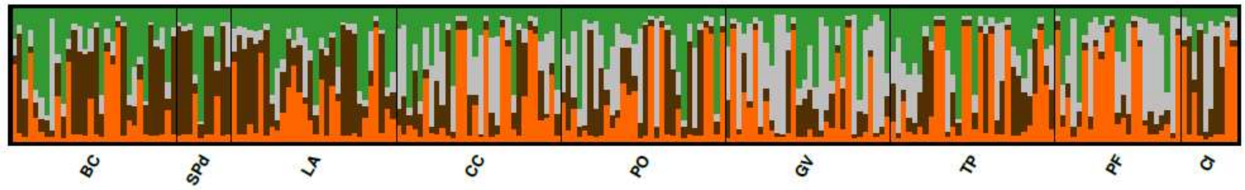


(b)

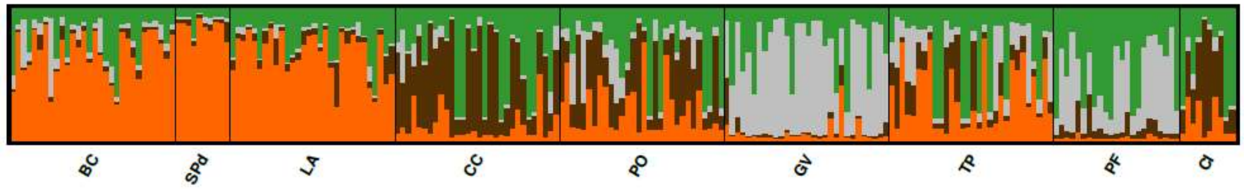


Figure S9. STRUCTURE results from Texas-Louisiana subset. (a) Without the LOCPRIOR model, ΔK method identified K = 4 and ln Pr(X|K) increased until an initial plateau at K = 4. Cluster assignments were roughly symmetric across samples, indicative of a lack of population structure (Pritchard et al., 2003). (b) With the LOCPRIOR model, ΔK method again identified K = 4 and ln Pr(X|K) again increased until an initial plateau at K = 4. Results indicated separation into southern, central, and northern clusters, plus a seemingly non-informative fourth cluster that covers the entire distribution range (green color in figure). Refer to Supporting information Table S1 for site codes.

MOLECULAR ECOLOGY

(a) (b) (c) (d)

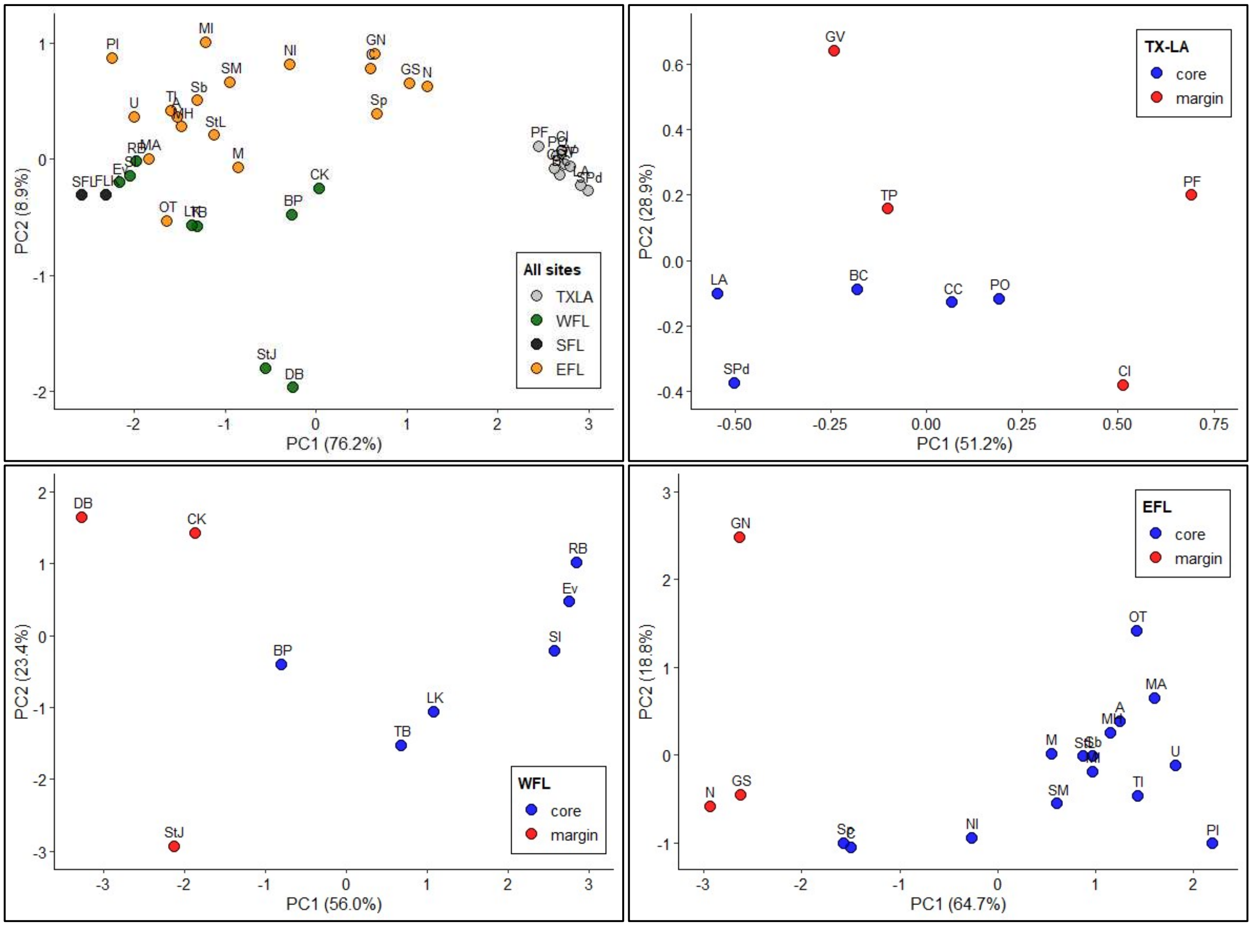


Figure S10. Principal coordinates analysis (PCoA) for (a) the entire USA *Avicennia germinans* distribution range, (b) Texas-Louisiana (TX-LA), (c) West Florida (WFL), and (d) East Florida (EFL). Panel a: South Florida (SFL) collection sites in black, WFL in green, EFL in orange, and TX-LA in grey. Panel b-d: Range core sites in blue and margin sites in red. Refer to Supporting information Table S1 for site codes. NOTE: In panel b, inconsistent with STRUCTURE results, PCoA indicated further separation between northern Texas (code: GV) and Louisiana (code: PF, CI).

## References

Cavanaugh, K. C., Osland, M. J., Bardou, R., Hinojosa-Arango, G., López-Vivas, J. M., Parker, J. D., & Rovai, A. S. (2018). Sensitivity of mangrove range limits to climate variability. *Global Ecology and Biogeography*, *27*(8), 925–935. doi: 10.1111/geb.12751

Cerón-Souza, I., Bermingham, E., McMillan, W. O., & Jones, F. A. (2012). Comparative genetic structure of two mangrove species in Caribbean and Pacific estuaries of Panama. *BMC Evolutionary Biology*, *12*, 205. doi: 10.1186/1471-2148-12-205

Cerón-Souza, I., Rivera-Ocasio, E., Funk, S. M., & McMillan, W. O. (2006). Development of six microsatellite loci for black mangrove (Avicennia germinans). *Molecular Ecology Notes*, *6*(3), 692–694. doi: 10.1111/j.1471-8286.2006.01312.x

Chapuis, M. P., & Estoup, A. (2007). Microsatellite null alleles and estimation of population differentiation. *Molecular Biology and Evolution*, *24*(3), 621–631. doi: 10.1093/molbev/msl191

Giri, C., Ochieng, E., Tieszen, L. L., Zhu, Z., Singh, A., Loveland, T., … Duke, N. (2011). Status and distribution of mangrove forests of the world using earth observation satellite data. *Global Ecology and Biogeography*, *20*(1), 154–159. doi: 10.1111/j.1466-8238.2010.00584.x

Guo, H., Zhang, Y., Lan, Z., & Pennings, S. C. (2013). Biotic interactions mediate the expansion of black mangrove (Avicennia germinans) into salt marshes under climate change. *Global Change Biology*, *19*(9), 2765–2774. doi: 10.1111/gcb.12221

Janes, J. K., Miller, J. M., Dupuis, J. R., Malenfant, R. M., Gorrell, J. C., Cullingham, C. I., & Andrew, R. L. (2017). The K = 2 conundrum. *Molecular Ecology*, *26*, 3594–3602. doi: 10.1111/mec.14187

Jost, L. (2008). GST and its relatives do not measure differentiation. *Molecular Ecology*, *17*(18), 4015–4026. doi: 10.1111/j.1365-294X.2008.03887.x

Meirmans, P. G., & Hedrick, P. W. (2011). Assessing population structure: FST and related measures. *Molecular Ecology Resources*, *11*(1), 5–18. doi: 10.1111/j.1755-0998.2010.02927.x

Mori, G. M., Zucchi, M. I., Sampaio, I., & Souza, A. P. (2010). Microsatellites for the mangrove tree Avicennia germinans (Acanthaceae): Tools for hybridization and mating system studies. *American Journal of Botany*, *97*(9), 79–81. doi: 10.3732/ajb.1000219

Nettel, A., Rafii, F., & Dodd, R. S. (2005). Characterization of microsatellite markers for the mangrove tree Avicennia germinans L. (Avicenniaceae). *Molecular Ecology Notes*, *5*(1), 103–105. doi: 10.1111/j.1471-8286.2004.00851.x

Osland, M. J., Feher, L. C., Griffith, K. T., Cavanaugh, K. C., Enwright, N. M., Day, R. H., … Rogers, K. (2017). Climatic controls on the global distribution, abundance, and species richness of mangrove forests. *Ecological Monographs*, *87*(2), 341–359. doi: 10.1002/ecm.1248

Pritchard, J. K., Wen, W., & Falush, D. (2003). *Documentation for STRUCTURE Software: Version 2*. Retrieved from http://web.stanford.edu/group/pritchardlab/software/structure_v.2.3.1/documentation.pdf

QGIS Development Team. (2017). *QGIS Geographic Information System*. Website: qgis.osgeo.org: Open Source Geospatial Foundation Project.

Sherrod, C. L., & McMillan, C. (1981). Black mangrove, Avicennia germinans, in Texas: past and present distribution. *Contributions in Marine Science*, *24*, 115–131.

van Oosterhout, C., Hutchinson, W. F., Wills, D. P. M., & Shipley, P. (2004). MICRO-CHECKER: Software for identifying and correcting genotyping errors in microsatellite data. *Molecular Ecology Notes*, *4*(3), 535–538. doi: 10.1111/j.1471-8286.2004.00684.x
